# Comparison of calibration methods in the analysis of 2013 Brazilian National Health Survey data

**DOI:** 10.1590/1980-549720250005

**Published:** 2025-02-24

**Authors:** Juliana Sena de Souza, Márcia Helena Barbian, Rodrigo Citton Padilha dos Reis

**Affiliations:** I Universidade Federal do Rio Grande do Sul, Graduate Program in Epidemiology – Porto Alegre (RS), Brazil.; II Universidade Federal do Rio Grande do Sul, Department of Statistics – Porto Alegre (RS), Brazil.

**Keywords:** Population forecast, Sampling studies, Epidemiologic studies, Population studies in public health, Statistics as topic

## Abstract

**Objective:**

This study aims to compare calibration methods for weights in the subsample of Laboratory Exams from the 2013 Brazilian National Health Survey (PNS), seeking to assess their representativeness and precision.

**Methods:**

Two alternative proposals for constructing calibrated weights were performed based on post-stratification and raking methods. A comparison between the weights provided for the Laboratory Exams subsample and the two suggested weights was conducted through parameter estimates using the 2013 PNS subsample data. Additionally, seven measures were used to assess the performance of the proposed weighting systems.

**Results:**

The alternative post-stratification and raking weights produced generalizable estimates for the target population of the 2013 PNS, while the original weights did not. The alternative methods showed similar performance to the original method, with a slight advantage for raking in some evaluation measures.

**Conclusion:**

It is recommended that basic design weights be documented and included in the public-use data files of the PNS. Furthermore, it is suggested to cross-reference information between the sample and subsample of the 2013 PNS to enable the exploration of methods such as data imputation, aiming to obtain more accurate and representative estimates. These improvements are essential to ensure the quality and usefulness of PNS data in epidemiological and public health studies.

## INTRODUCTION

The Brazilian National Health Survey (*Pesquisa Nacional de Saúde* – PNS), conducted by the Ministry of Health in collaboration with Fundação Oswaldo Cruz (Fiocruz) and the Brazilian Institute of Geography and Statistics (*Instituto Brasileiro de Geografia e Estatística* – IBGE), represents the most extensive study ever undertaken in Brazil on health conditions and their determinants^1^. It enables the assessment of access to diagnosis and healthcare services^2^ while generating comprehensive data on the lifestyle patterns of the Brazilian population.

A significant innovation in the 2013 PNS was the inclusion of biological material collection (blood and urine samples) from a subsample comprising 25% of participants who completed the initial phase of the survey. This advancement enabled laboratory analyses and studies on the prevalence of anemia, total cholesterol levels, kidney failure, diabetes, and other health conditions, along with their associated factors within the Brazilian population^3–7^. Furthermore, this marked the first time such a study was conducted on a nationwide scale^8^.

Despite the innovative approach, challenges in fieldwork led to a loss of over 20% of the subsample designated for laboratory testing, resulting in 8,952 participants providing biological material. To address these losses and ensure the validity of the results, a post-stratification weighting method was applied for data analysis^9^.

Alternative techniques to post-stratification have been proposed in the literature, with raking^10^ and two-phase sampling calibration^11,12^ emerging as prominent methods. The evaluation of estimator performance, taking into account sampling weights and other design features, remains a critical area of focus in research on complex survey sampling designs^13,14^.

In this context, the study aimed to compare various weight calibration methods to address distortions in the subsample of laboratory tests from the 2013 PNS and to enhance the accuracy and reliability of the resulting estimates. Performance evaluation measures^15^ were employed to identify the most effective calibration strategies, ensuring the optimal use of the data collected by the PNS and contributing to a more comprehensive understanding of public health in Brazil.

## METHODS

### Calibration in the subsample of Laboratory Tests of the 2013 PNS

Given that the Laboratory Tests subsample of the 2013 PNS was designed based on the distance between the sector selected (for the first phase of the survey) and municipalities with large populations (those with 80,000 or more inhabitants) within the state of the sector^9^, it was anticipated that a weighting system for a two-phase design ( $\left\{d_{k}^{*};k\in S_{2}\right\}$ , where *S*_2_ represents the Laboratory Tests subsample) would be provided to statisticians, epidemiologists, and other researchers using the 2013 PNS data. This information could be utilized to derive estimates using the double expansion estimator, as outlined in Equation 3 of Supplementary Material 1, or even by employing the calibration estimator. This would enable data users to construct calibrated weighting systems based on the basic weights of the two-phase design $d_{k}^{*}$ .

On the other hand, "post-stratification" weights were provided alongside the data from the Laboratory Tests subsample (*weight_lab*). These weights, denoted as $W_{k}^{\left(lab\right)}$ , are defined in the article describing the subsample methodology^9^. In calculating the "post-stratification" weights, data from the 60,202 participants selected for individual interviews during the first phase of the 2013 PNS were considered. The following auxiliary variables were used to define the strata: gender (two levels: male and female); age (four groups: 18 to 29 years, 30 to 44 years, 45 to 59 years, and 60 years old or older); race/color (four categories: white, black, brown, and other); level of education (three levels: incomplete elementary school, completed elementary school and/or incomplete high school, and completed high school or higher); and geographic macroregion (five levels: South, Southeast, Central-West, North, and Northeast). This resulted in a total of 480 post-strata. The "post-stratification" weights were then defined by^9^ (Equation 1):


(1) $$w_{k}^{\left(lab\right)}=\frac{N_{h}}{n_{h}}\times \frac{\sum n_{h}}{\sum N_{h}},for\;k\;belonging\;to\;stratum\;h\text{,}$$

Where:

*N_h_*: the number of residents selected from the 2013 PNS in each stratum,

*h* and *n_h_*: the number of corresponding observations in the Laboratory Tests subsample^9^.

It is worth noting that, in the notation for a two-phase design, *N_h_* and *n_h_* would be represented as *n_1h_* and *n_2h_*, respectively.

Concerning the construction and application of the proposed "post-stratification" weights, three key observations are highlighted:

- Although it is not possible to confirm with certainty, the quantity $\sum n_{h}/\sum N_{h}$ appears to approximate *n*_1_/*n*_2_ = 8,952/60,202, representing the "sampling fraction" relative to the sample from the first phase of the 2013 PNS. Additionally, it is noted that the weights (1) do not seem to be genuine post-stratification weights, as they fail to incorporate the basic design weights;
- The estimates produced using data from the Laboratory Tests subsample and weights $W_{k}^{\left(lab\right)}$ are generalizable to the 2013 PNS sample of 60,202 participants, but not to the target population of the PNS, which comprises adults residing in permanent private households;
- When analyzing data from the Laboratory Tests subsample, the weights defined in (1) are applied in conjunction with the subsample design specification. Typically, the subsample is treated as having unequal inclusion probabilities, where the basic weights are represented by $W_{k}^{\left(lab\right)}$ . This approach leads to variance calculations for the estimators based on these weights $W_{k}^{\left(lab\right)}$ .

Regarding this last observation, it is suggested that the variance of the estimators should be calculated using the weights *d_k_*, or a weighting system that closely approximates this expression. Additional details on the fundamental definitions of sampling designs, the formulation of basic weights, and the construction of calibrated weighting systems can be found in Supplementary Material 1.

### Alternative calibration methods

Since the variables used to construct the weights $W_{k}^{\left(lab\right)}$ are available in the 2013 PNS microdata files for both the full sample of 60,202 participants and the Laboratory Tests subsample of 8,952 participants, an alternative method for analyzing the data from the PNS 2013 subsample is proposed. This approach involves the following design and weight construction: it is assumed that the Laboratory Tests subsample was selected through simple random sampling without replacement from the first-phase sample of the 2013 PNS. Accordingly, the basic weights take the form $d_{k}=n_{1}/n_{2}=60,202/8,952\approx 6.72$ for all 8,952 participants in the second phase of the PNS 2013. Post-stratification weights were then constructed using the same auxiliary variables as the original weights $W_{k}^{\left(lab\right)}$ but adopting the "Population projection" variable (available in the PNS 2013 microdata as variable *V00282*) as the reference. Using this projection, the total population of Brazilian adults is estimated at 145,572,210. The post-stratification weights were calculated using Equation 2:


(2) $$w_{k}^{PS_{AAS}}=d_{k}\frac{N_{h}}{{\hat{N}}_{h,\pi }}=\frac{n_{1}}{n_{2}}\times \frac{N_{h}}{{\hat{N}}_{h,\pi }}=\frac{n_{1}}{n_{2}}\times \frac{N_{h}}{n_{2h}}\times \frac{n_{2}}{n_{1}}=\frac{N_{h}}{n_{2h}},$$

for *k* belonging to stratum *h*, where *n*_2*h*_ corresponds to the number of participants in stratum *h* in subsample *S_2_*. The second-to-last equality in (2) is justified because ${\hat{N}}_{h,\pi }=\sum_{K\in S_{2}\Omega U_{h}}d_{k}=n_{2h}\times \left(n_{1}/n_{2}\right)$ , given that we assume *d_k_* = *n*_1_/*n*_2_.

Note that expression (2) is identical to the first equation in the article on the methodology of the Laboratory Tests subsample^9^, with the distinction that, in (2), *N_h_* represents the number of residents in each stratum *h* in the Brazilian population, rather than the number of residents selected from the 2013 PNS in each stratum *h*. Thus, we propose a weighting system designed to produce estimates generalizable to the target population of the 2013 PNS.

A second alternative for creating a calibrated weights system, still assuming that the basic weights correspond to simple random sampling, was implemented using the method known as raking. This iterative process involves sequentially post-stratifying each set of variables and repeating the procedure until the weights converge to a stable solution^16^. Raking enables the use of multiple grouping variables without requiring the construction of a complete cross-classification. The auxiliary variables used to construct these weights were the same as those used for the weights $W_{k}^{\left(PS_{AAS}\right)}$ , and the "Population projection" variable was also incorporated. The weights generated through the raking method will be denoted as $W_{k}^{\left(rake_{AAS}\right)}$ .

### Ethical aspects

This study utilized publicly available and anonymized data from the IBGE PNS. Consequently, approval from a Research Ethics Committee was not required.

### Statistical analysis

To evaluate the underrepresentation and overrepresentation of the groups defined by the auxiliary variables (used to construct the calibrated weights), population projections (based on the 2013 PNS data) were assumed to represent the true population values. The relative frequencies estimated using the three weight systems $W_{k}^{\left(lab\right)}$ , $W_{k}^{\left(PS_{AAS}\right)}$ , and $W_{k}^{\left(rake_{AAS}\right)}$ were then compared with the corresponding designs previously described.

To compare the proposed calibrated weights with the weights provided for the Laboratory Tests subsample, population parameters were estimated for selected variables of interest, as outlined in Table 1. Variables with codes beginning with the letter "Z" were collected during the Laboratory Tests phase of the 2013 PNS. In contrast, variables with codes starting with "Q" or "J" were collected during the first phase of the 2013 PNS and are available for the full sample of 60,202 participants.

**Table 1:** Characteristics of interest in the Laboratory Tests subsample of the 2013 PNS selected for calibration estimate evaluation.

Variable Code	Variable Description	Parameter Type
Q030	Diabetes (*yes, no, only during pregnancy*)	Total prevalence
Q002	Hypertension (*yes, no, only during pregnancy*)	Total prevalence
Q060	High cholesterol (*yes, no*)	Total prevalence
Q063	Heart disease [heart attack, angina, heart failure, or other] (*yes, no*)	Total prevalence
Q068	Stroke or cerebral hemorrhage (*yes, no*)	Total prevalence
Q120	Cancer (*yes, no*)	Total prevalence
Q124	Chronic kidney failure – CKD (*yes, no*)	Total prevalence
J058	Dengue (*yes, no*)	Total prevalence
Z025	Creatinine (mg/dL)	Total and average
Z031	Total cholesterol (mg/dL)	Total and average
Z034	Glycated hemoglobin – HbA1c (%)	Total and average

To evaluate the estimates generated using the proposed weighting systems in $W_{k}^{\left(PS_{ASS}\right)}$ and $W_{k}^{\left(rake_{ASS}\right)}$ , the seven measures outlined in Supplementary Material 2 were calculated. The analyses were conducted using the R software^17^, version 17, along with the survey package^18^ to account for the sampling design. The R code specifying the design object (svydesign) used in the analyses is provided in Supplementary Material 3.

## RESULTS

### Weight distribution

**Figure 1:**
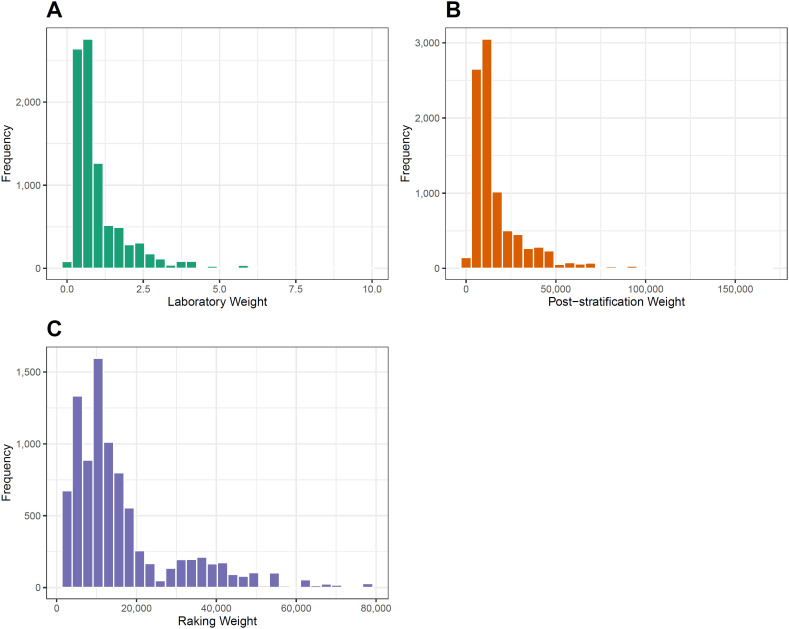
Distribution of weights (A) post-stratification weights provided with the Laboratory Tests subsample data from the 2013 PNS; (B) post-stratification weights; and (C) raking weights constructed from the population projection – Laboratory Tests subsamples, 2013 PNS.

The weight distributions of $W_{k}^{\left(lab\right)}$ and $W_{k}^{\left(PS_{AAS}\right)}$ exhibit a similar shape; however, the values each weight system assumes differ significantly, reflecting the distinct objectives of each system. On one hand, $W_{k}^{\left(lab\right)}$ aims to represent the sample from the first phase of the 2013 PNS (Figure 1A). On the other hand, $W_{k}^{\left(PS_{AAS}\right)}$ is designed to represent the target population of the survey, namely, Brazilian adults living in permanent private households (Figure 1B). The distribution of raking weights shows a distinct shape compared to the other weight distributions of $W_{k}^{\left(lab\right)}$ and $W_{k}^{\left(PS_{AAS}\right)}$ , but the values are similar to those of $W_{k}^{\left(PS_{AAS}\right)}$ . Therefore, the raking weights $W_{k}^{\left(rake_{AAS}\right)}$ also yield generalizable estimates for the target population of the 2013 PNS (Figure 1C).

### Representation of post-strata

The weights $W_{k}^{\left(lab\right)}$ produce estimates of the proportions for the auxiliary variables that are close to the population projections (Figure S1, Supplementary Material). Generally, the estimates based on $W_{k}^{\left(lab\right)}$ show a difference (absolute error) of no more than 0.15%, except for the categories of brown race/color (0.22%), other race/color (0.42%), and the Northeast region (-0.26%). The estimates obtained using the weights $W_{k}^{\left(PS_{AAS}\right)}$ also present an absolute error typically no greater than 0.15%, with the exceptions of white race/color (0.21%), brown race (-0.20%), and other race/color (0.43%). For the weights calibrated using the raking method, $W_{k}^{\left(rake_{AAS}\right)}$ , the estimates of the proportions for the auxiliary variables align precisely with the population projections. Similar results were obtained when comparing the estimates of the population totals for the auxiliary variables (Table S1, Supplementary Material 4). While the relative error (RE) for $W_{k}^{\left(lab\right)}$ was considered, it became evident that the population estimates derived from these weights diverge significantly from the population projections, resulting in an exceptionally high RE — therefore, these results were not presented.

### Calibration assessment measures

The seven evaluation measures for the calibration methods are presented in Table 2. The mean absolute calibration RE was *M*_1_ = 2.16 for the post-stratification method and *M*_1_ = 0 for raking. These results were anticipated, as the estimated totals for the auxiliary variables were generally lower than the population totals. The mean coefficients of variation for the totals of the auxiliary variables *M*_2_ = 0% indicate that the estimates produced by both methods are unbiased.

**Table 2:** Performance measures of post-stratification and raking calibration methods – Laboratory Tests subsample, 2013 PNS.

Evaluation Measure	$W_{k}^{\left(PS_{AAS}\right)}$	$W_{k}^{\left(rake_{AAS}\right)}$
M1: Mean of absolute relative calibration error	2.16	0.00
M2: Mean of the coefficient of variation (CV) of the totals of auxiliary variables	0.00%	0.00%
M3: Proportion of extreme weights relative to 1/3	19.02%	18.81%
M4: Proportion of extreme weights relative to 3	4.39%	3.24%
M5: Coefficient of variation of the weights *g*	91.37%	83.94%
M6: Distance *X* ^2^ between *w* and *d*	13,440.07	11,447.09
M7: Average efficiency of calibration estimates	1.005	1.018

The measures of *M*_3_ and *M*_4_ (proportion of extreme weights) indicate the presence of extreme *g* weights for both the post-stratification and raking methods. This outcome was anticipated, considering the difference between the design weights $W_{k}^{\left(PS_{AAS}\right)}$ and $W_{k}^{\left(rake_{AAS}\right)}$ (*M*_6_ = 13,440.07 for post-stratification and *M*_6_ = 11,447.09 for raking). The coefficient of variation for the *g* weights further reflects this characteristic of the calibrated weight construction and was high for both methods (*M*_5_ = 91.37% for post-stratification and *M*_5_ = 83.94% for raking), with a slight advantage observed for the raking method.

The average efficiency of the estimates (*M*_7_) by the alternative calibration methods for the set of variables presented in Table 1 indicates a slight advantage of the post-stratification method over raking.

### Accuracy of estimates of parameters of interest

Table 3 presents the estimates for the parameters related to the characteristics of interest (listed in Table 1), along with the coefficients of variation obtained from the three calibrated weighting systems. It can be observed that the point estimates (totals, prevalences, and means) produced by the three methods are generally very similar. The exception is the estimate of population totals from the post-stratification weights of the Laboratory Tests subsample itself, as indicated by the weights $W_{k}^{\left(lab\right)}$ . As previously noted, these results are not generalizable to the target population of the 2013 PNS. Lastly, the estimates of the coefficients of variation for the estimates show greater precision than those of the alternative calibration methods proposed in this study. This outcome was anticipated, as the estimation of the standard error of the estimates incorporates aspects of the assumed sampling design.

**Table 3:** Estimated totals and prevalences (%)‡, and coefficient of variation (CV%) of the characteristics of interest obtained from the three calibrated weight systems — Laboratory Tests subsample, 2013 PNS.

Characteristics of interest	$W_{k}^{\left(lab\right)}$ (N = 8,952)		$W_{k}^{\left(PS_{AAS}\right)}$ (N = 144,908,922)		$W_{k}^{\left(rake_{AAS}\right)}$ (N = 145,572,210)	
	Estimate (%)	CV%	Estimate (%)	CV%	Estimate (%)	CV%
*Diabetes*	573 (7.69)	5.04	9,275,930 (7.7)	4.76	9,266,527 (7.65)	4.85
*Hypertension*	1,930 (25.91)	2.53	31,236,815 (25.92)	2.18	31,171,025 (25.75)	2.25
*High cholesterol*	1,160 (15.57)	3.42	18,773,491 (15.58)	3.2	18,842,610 (15.56)	3.25
*Heart disease*	370 (4.96)	6.15	5,971,338 (4.95)	5.83	6,092,130 (5.03)	5.98
*Stroke*	146 (1.96)	11.57	2,367,716 (1.96)	10.47	2,266,318 (1.87)	10.3
*Cancer*	158 (2.11)	9.87	2,540,009 (2.11)	9.45	2,549,079 (2.11)	9.57
*CKD*	111 (1.48)	12.04	1,785,325 (1.48)	11.78	1,815,305 (1.5)	11.73
*Dengue*	1,406 (18.88)	3.06	22,735,194 (18.86)	2.9	22,732,548 (18.78)	2.91
*Creatinine (Mean, mg/dL)*	0.881	0.44	0.881	0.38	0.882	0.44
*Total cholesterol (Mean, mg/dL)*	185.031	0.31	185.01	0.29	184.815	0.29
*HbA1c (Mean, %)*	5.513	0.23	5.513	0.22	5.51	0.23

‡ For the characteristics Creatinine, Total Cholesterol, and HbA1c, both the mean and CV were estimated.

To evaluate the performance of the calibration methods in estimating population subgroups, estimates of diabetes prevalence were obtained based on several characteristics of interest (Table 4). Once again, it can be observed that the point estimates are very similar across the three weighting systems, with the apparent advantage of the alternative calibration methods being seen in the precision of the estimates. The 95% confidence intervals for the prevalence of diabetes in population subgroups, produced by the post-stratification $\left(W_{k}^{\left(PS_{AAS}\right)}\right)$ and raking $\left(W_{k}^{\left(rake_{AAS}\right)}\right)$ methods, are slightly narrower than those produced by the weight $W_{k}^{\left(lab\right)}$ .

**Table 4:** Prevalence of diabetes (%) and 95% confidence interval (95%CI) by population subgroups obtained from the three calibrated weighting systems – Subsample of Laboratory Tests, PNS 2013.

Characteristics		$W_{k}^{\left(lab\right)}$		$W_{k}^{\left(PS_{AAS}\right)}$		$W_{k}^{\left(rake_{ASS}\right)}$	
		%	95%CI	%	95%CI	%	95%CI
Age							
	18 to 29 years	1.4	0.5–2.4	1.4	0.6–2.3	1.3	0.5–2.0
	30 to 44 years	2.4	1.6–3.3	2.4	1.6–3.2	2.4	1.6–3.1
	45 to 59 years	10.8	9.1–12.5	10.8	9.2–12.5	11.1	9.4–12.8
	≥60 years	18.2	15.9–20.5	18.2	16.0–20.5	17.9	15.6–20.2
Race/color							
	White	7.3	6.2–8.4	7.3	6.3–8.3	7.3	6.3–8.4
	Black	9.6	6.8–12.5	9.7	7.1–12.2	9.7	6.9–12.4
	Brown	7.2	6.2–8.3	7.2	6.2–8.2	7.1	6.1–8.2
	Other	6.0	1.1–10.8	45.5	2.0–8.9	4.6	1.1–8.0
Body Mass Index – BMI (kg/m²)							
	Underweight/normal (BMI<25)	4.2	3.4–4.1	4.2	3.4–5.0	4.3	3.4–5.2
	Overweight (25≤BMI<30)	7.5	6.2–8.7	7.5	6.2–8.7	7.2	6.0–8.4
	Obesity (BMI≥30)	13.5	11.4–15.5	13.5	11.5–15.4	13.5	11.6–15.5
Health Insurance							
	Yes	7.1	5.8–8.4	7.1	5.8–8.3	7.0	5.8–8.3
	No	7.6	6.8–8.5	7.7	6.8–8.5	7.6	6.7–8.4

## DISCUSSION

The 2013 PNS included the collection of a subsample of laboratory tests, which represents a significant contribution to studies on the health of the Brazilian population. Sampling techniques suggest that a two-phase design could have been employed in the 2013 PNS subsample to construct both basic and calibrated weighting systems. However, challenges in collecting the second-phase sample led to the non-disclosure of the design weights alongside the microdata of the Laboratory Tests subsample. In the absence of basic sampling weights, the managers of the subsample data provided calibrated weights using the post-stratification method.

This study proposed two alternative calibration methods based on post-stratification and raking. The weighting systems obtained from these methods demonstrated performance comparable to the weighting system available with the Laboratory Test data. Notably, it is important to highlight that the estimates derived from the proposed weighting systems are generalizable to the target population of the 2013 PNS: the Brazilian adult population living in private households.

Another aspect to emphasize is that the two proposed calibration methods demonstrated greater precision for the estimates considered in this study. A possible explanation for this behavior is that the methods incorporated important aspects, albeit presumed, of the sampling survey design, which contributed to the accurate calculation of the variance estimates for the parameters of interest.

When using measures to assess the performance of the calibration methods, the two suggested weighting systems performed well, showing an advantage over the raking-based weights. Some previous studies have reached similar conclusions^19–22^. However, it is important to note that measures comparing the calibrated weights to the "pre-calibrated" weights reveal a substantial difference between the two sets of weights. This behavior is likely due to the assumption of a simple random sampling design for the first-phase sample of the 2013 PNS data from the Laboratory Tests subsample, while the post-strata were constructed based on projections of the Brazilian population.

As observed, the Laboratory Tests subsample of the 2013 PNS could be considered the result of a two-phase sampling design^16^. However, the selection probabilities (or, equivalently, the basic sampling weights) were not made available in the Laboratory Tests microdata file, limiting the potential benefits of a two-phase design. The availability of the basic design weights for the Laboratory Tests subsample would enable statisticians and epidemiologists to construct calibrated weighting systems, thereby improving the performance of calibration estimators.

Regarding the estimation of population totals (*e.g*., the total number of individuals with diabetes), the two methods proposed in this article provide estimates for the target population of the 2013 PNS, allowing the results to be generalized to the Brazilian adult population. The weighting system provided with the data from the Laboratory Tests subsample produced estimates specific to the 2013 PNS sample. While it is possible to obtain estimates for the target population of the PNS indirectly by multiplying the sample proportion by the population size, it is preferable for the methods, along with the appropriate software, to provide these estimates directly to minimize errors in the interpretation of the results.

The approach adopted in this article involved weighting through calibration methods. Some limitations of this approach are inherent to the method itself, such as the application of weights to more complex estimates, like regression coefficients; the difficulty in assessing the standard errors of weighted estimates, and the decisions involved in constructing weighting systems^23^. However, it is recommended that weighted estimates be used in preference to unweighted estimates, particularly when working with data from population surveys that employ complex sampling designs. Another limitation of this work was the assumption of certain aspects of the design used to collect data from the 2013 PNS Laboratory Tests subsample. As emphasized in the literature, analyses should account for the relevant aspects of the sampling plan^14^. Making such details available for the PNS Laboratory Tests subsample would enhance researchers’ ability to estimate population quantities with greater precision.

We conclude with two suggestions for survey designers and managers of the Brazilian National Health Surveys that will be used by other researchers. First, all weights related to the design of the PNS should be thoroughly documented and included in the public data files. This would enable users to construct estimates based on either the basic or calibrated weights, utilizing their own auxiliary variables. Accurate precision of the estimates could then be achieved through the proper use of data analysis software designed for complex sampling plans.

Our second recommendation pertains to the keying of information obtained from the sample and subsample (of Laboratory Tests) of the 2013 PNS. Properly documenting this information would facilitate the application of methods such as data imputation to obtain more accurate estimates. Given the significant relevance of these data for research in epidemiology and public health, it is essential that the most appropriate methods be employed in their analysis.
