# Supplementary material for: Comparison of calibration methods in the analysis of 2013 Brazilian National Health Survey data
Source: Rev Bras Epidemiol. 2025 Feb 24;28:e250005. doi: 10.1590/1980-549720250005 (PMC11849994; doi:10.1590/1980-549720250005)
Supplement: Translation

# Translation: Comparação de métodos de calibração na análise de dados da Pesquisa Nacional de Saúde de 2013

**DOI:** 10.1590/1980-549720250005.2

**Authors:** Juliana Sena de Souza, Márcia Helena Barbian, Rodrigo Citton Padilha dos Reis

**Affiliations:** I Universidade Federal do Rio Grande do Sul, Programa de Pós-Graduação em Epidemiologia – Porto Alegre (RS), Brasil.; II Universidade Federal do Rio Grande do Sul, Departamento de Estatística – Porto Alegre (RS), Brasil.

**Keywords:** Estimativas populacionais, Estudos de amostragem, Estudos epidemiológicos, Estudos populacionais em saúde pública, Métodos e procedimentos estatísticos

## Abstract

**Objetivo:**

Este estudo visa comparar métodos de calibração de pesos na subamostra de Exames Laboratoriais da Pesquisa Nacional de Saúde (PNS) 2013, buscando avaliar sua representatividade e precisão.

**Métodos:**

Duas propostas alternativas de construção de pesos calibrados foram realizadas com base nos métodos de pós-estratificação e *raking*. Uma comparação entre os pesos fornecidos para a subamostra de Exames Laboratoriais e os dois pesos sugeridos foi realizada por meio de estimativas de parâmetros de interesse utilizando os dados da subamostra da PNS 2013. Além disso, foram utilizadas sete medidas para avaliar o desempenho dos sistemas de pesos propostos.

**Resultados:**

Os pesos de pós-estratificação e *raking* alternativos produziram estimativas generalizáveis para a população-alvo da PNS 2013, enquanto os pesos originais não. Os métodos alternativos apresentaram desempenho similar ao método original, com pequena vantagem para o *raking* em algumas medidas de avaliação.

**Conclusão:**

Recomenda-se que os pesos básicos de delineamento sejam documentados e incluídos nos arquivos de dados de uso público da PNS. Além disso, sugere-se o chaveamento das informações entre a amostra e a subamostra da PNS 2013 para possibilitar a exploração de métodos como a imputação de dados, visando obter estimativas mais precisas e representativas. Essas melhorias são essenciais para garantir a qualidade e utilidade dos dados da PNS em estudos epidemiológicos e de saúde coletiva.

## INTRODUÇÃO

A Pesquisa Nacional de Saúde (PNS), conduzida pelo Ministério da Saúde em parceria com a Fundação Oswaldo Cruz (Fiocruz) e o Instituto Brasileiro de Geografia e Estatística (IBGE), é a pesquisa mais ampla já realizada no país sobre a situação de saúde e seus determinantes^1^, permitindo o dimensionamento do acesso ao diagnóstico e à atenção à saúde^2^ e a produção de dados dos estilos de vida da população brasileira.

Um grande diferencial da PNS de 2013 foi a inclusão da coleta de material biológico (amostras de sangue e urina dos participantes) para uma subamostra de 25% dos indivíduos que responderam à primeira fase da pesquisa, o que permitiu a realização de exames laboratoriais e a elaboração de estudos referentes à prevalência de anemia, colesterol total, insuficiência renal e diabetes, além de outros temas e fatores associados da população brasileira^3–7^. Além disso, esse estudo é de abrangência nacional, o que nunca havia acontecido até então^8^.

No entanto, devido a dificuldades no trabalho de campo, houve uma perda superior a 20% na subamostra de indivíduos indicados para a realização dos exames laboratoriais, resultando em 8.952 participantes que forneceram material biológico. Devido a essas perdas, o método de ponderação de pós-estratificação foi proposto para a análise dos dados e para garantir a validade dos resultados^9^.

Técnicas alternativas à pós-estratificação foram propostas na literatura, entre as quais se destacam o *raking* ^10^ e a calibração de amostragem em duas fases^11,12^. A avaliação do desempenho dos estimadores considerando pesos amostrais e demais características do delineamento é uma questão recorrente no campo de pesquisa de levantamentos por amostragem de planos complexos^13,14^.

Neste cenário, o objetivo do presente estudo foi comparar diferentes métodos de calibração de pesos, buscando não apenas corrigir as distorções da subamostra de Exames Laboratoriais da PNS 2013, mas também melhorar a precisão e a confiabilidade das estimativas. Foram utilizadas medidas de avaliação de desempenho^15^ para identificar a estratégia mais eficaz de calibração dos pesos para garantir que os dados coletados pela PNS possam ser plenamente aproveitados, contribuindo para uma compreensão mais profunda da saúde pública no Brasil.

## MÉTODOS

### Calibração na subamostra de Exames Laboratoriais da PNS 2013

Tendo em vista que a subamostra de Exames Laboratoriais da PNS 2013 foi delineada com base na informação de distância entre o setor selecionado para a PNS 2013 (primeira fase) e os municípios de grande porte populacional (aqueles municípios com 80 mil habitantes ou mais) dentro da UF do setor^9^, esperava-se que um sistema de pesos de um delineamento em duas fases ( $\left\{d_{k}^{*};k\in S_{2}\right\}$ , em que *S*_2_ representa a subamostra de Exames Laboratoriais) fosse fornecido aos estatísticos, epidemiologistas e outros pesquisadores que fazem uso dos dados da PNS 2013. Tal informação poderia ser utilizada para a obtenção de estimativas a partir do estimador de expansão dupla, disponível na Equação 3 do Material Suplementar 1, ou ainda, com base no estimador de calibração, permitindo aos usuários dos dados a construção de sistemas de pesos calibrados a partir dos pesos básicos do delineamento em duas fases $d_{k}^{*}$ .

Por outro lado, pesos "pós-estratificação" foram fornecidos em conjunto com os dados da subamostra de Exames Laboratoriais (*peso_lab*). Denotaremos os pesos fornecidos para a subamostra de Exames Laboratoriais por $W_{k}^{\left(lab\right)}$ , cuja definição pode ser encontrada no artigo de metodologia da subamostra^9^. No cálculo dos pesos "pós-estratificação", foram considerados os dados dos 60.202 participantes selecionados para as entrevistas individuais na primeira fase da PNS 2013. As seguintes variáveis auxiliares foram consideradas para a composição dos estratos: sexo (dois níveis: masculino e feminino); idade (4 faixas etárias: 18 a 29 anos, 30 a 44 anos, 45 a 59 anos e 60 anos ou mais); raça/cor (quatro níveis: branca, preta, parda e outros); grau de instrução (três níveis: fundamental incompleto, fundamental completo e/ou ensino médio incompleto e ensino médio completo ou mais) e macrorregião geográfica (cinco níveis: Sul, Sudeste, Centro-Oeste, Norte e Nordeste), resultando em um total de 480 pós-estratos. Os pesos "pós-estratificação" foram então definidos por^9^ (Equação 1):


(1) $$w_{k}^{\left(lab\right)}=\frac{N_{h}}{n_{h}}\times \frac{\sum n_{h}}{\sum N_{h}},para\;k\;pertencente\;ao\;estrato\;h,$$

Em que:

*N_h_*: o número de moradores selecionados da PNS 2013 em cada estrato,

*h* e *n_h_*: o número de observações correspondentes na subamostra de Exames Laboratoriais^9^.

Note que, utilizando a notação de delineamentos em duas fases, *N_h_* e *n_h_* seriam denotados por *n*_1*h*_ e *n*_2*h*_, respectivamente.

Sobre a construção e utilização dos pesos "pós-estratificação" propostos, destacamos três observações:

- Não é possível saber ao certo, mas a quantidade $\sum n_{h}/\sum N_{h}$ parece ser aproximadamente *n*_1_/*n*_2_ = 8952/60.202, a "fração de amostragem" em relação à amostra da primeira fase da PNS 2013. Ainda, notamos que os pesos (1) não parecem ser pesos pós-estratificação genuínos, pois estes não levam em consideração os pesos básicos de delineamento;
- As estimativas produzidas com os dados da subamostra de Exames Laboratoriais e os pesos $W_{k}^{\left(lab\right)}$ são generalizáveis para a amostra da PNS 2013 de 60.202 participantes, e não para a população-alvo da PNS, composta por adultos residentes em domicílios particulares permanentes;
- Na análise de dados da subamostra de Exames Laboratoriais, o uso dos pesos definidos em (1) é feito em conjunto com a definição do delineamento da subamostra. Em geral, assume-se uma amostra com probabilidades desiguais de inclusão, em que os pesos básicos são dados pelos pesos $W_{k}^{\left(lab\right)}$ . Isso resulta em um cálculo da variância dos estimadores a partir desses pesos $W_{k}^{\left(lab\right)}$ .

Em relação a essa última observação, nós argumentamos que a variância dos estimadores deveria utilizar os pesos *d_k_*, ou um sistema de pesos que se aproxime de tal expressão. Maiores informações referente às definições básicas dos delineamentos por amostragem, definição dos pesos básicos e sistema de pesos calibrados estão disponíveis no Material Suplementar 1.

### Métodos de calibração alternativos

Como as variáveis utilizadas para a construção dos pesos $W_{k}^{\left(lab\right)}$ são disponibilizadas nos arquivos de microdados da PNS 2013, para a amostra de 60.202 participantes e para a subamostra de Exames Laboratoriais de 8.952 participantes, propomos como forma alternativa de análise dos dados da subamostra da PNS 2013 os seguintes delineamento e construção dos pesos: assumimos que a subamostra de Exames Laboratoriais foi selecionada por amostragem aleatória simples sem reposição da amostra da primeira fase da PNS 2013. Os pesos básicos assumem a forma $d_{k}=n_{1}/n_{2}=60.202/8.952\approx 6,72$ para todos os 8.952 participantes da segunda fase da PNS 2013. Pesos pós-estratificação foram construídos utilizando as mesmas variáveis auxiliares dos pesos $W_{k}^{\left(lab\right)}$ , porém adotando como referência a variável "Projeção da população", disponível nos microdados da PNS 2013 (variável *V00282*). Com essa projeção, o total de adultos brasileiros é estimado em 145.572.210. Os pesos pós-estratificação são calculados por Equação 2:


(2) $$w_{k}^{PE_{AAS}}=d_{k}\frac{N_{h}}{{\hat{N}}_{h,\pi }}=\frac{n_{1}}{n_{2}}\times \frac{N_{h}}{{\hat{N}}_{h,\pi }}=\frac{n_{1}}{n_{2}}\times \frac{N_{h}}{n_{2h}}\times \frac{n_{2}}{n_{1}}=\frac{N_{h}}{n_{2h}},$$

para *k* pertencente ao estrato *h* em que *n*_2*h*_ corresponde ao número de participantes no estrato *h* na subamostra *S*_2_. A penúltima igualdade em (2) se justifica, pois ${\hat{N}}_{h,\pi }=\sum_{K\in S_{2}\Omega U_{h}}d_{k}=n_{2h}\times \left(n_{1}/n_{2}\right)$ , uma vez que assumimos *d_k_* = *n*_1_/*n*_2_.

Note que a expressão (2) é idêntica à primeira equação do artigo de metodologia da subamostra de Exames Laboratoriais^9^, com a diferença que em (2) *N_h_* representa o número de moradores em cada estrato *h* na população brasileira, e não mais o número de moradores selecionados da PNS 2013 em cada estrato *h*. Dessa forma, propomos um sistema de pesos que produz estimativas generalizáveis para a população-alvo da PNS 2013.

Uma segunda alternativa na criação do sistema de pesos calibrados, ainda assumindo que os pesos básicos correspondem à amostragem aleatória simples, foi obtida por meio do método conhecido como *raking*. O processo envolve a pós-estratificação de cada conjunto de variáveis por vez e a repetição desse processo até que os pesos se estabilizem^16^. O *raking* permite que várias variáveis de agrupamento sejam usadas sem a necessidade de construir uma classificação cruzada completa. As variáveis auxiliares utilizadas para construção desses pesos foram as mesmas utilizadas para construir os pesos $W_{k}^{\left(PE_{AAS}\right)},$ , e a variável "Projeção da população" também foi utilizada. Denotaremos por $W_{k}^{\left(rake_{AAS}\right)}$ os pesos obtidos a partir do *raking*.

### Aspectos éticos

Este estudo utilizou dados de acesso públicos e anonimizados da PNS do IBGE, portanto, não foi necessária a aprovação do Comitê de Ética em Pesquisa.

### Análise estatística

Para avaliar a sub e sobre representação dos grupos das variáveis auxiliares (utilizadas para construção dos pesos calibrados), supondo que as projeções populacionais (a partir dos dados da PNS 2013) são os valores verdadeiros da população, foram comparadas as frequências relativas estimadas pelos três sistemas de pesos $W_{k}^{\left(lab\right)}$ , $W_{k}^{\left(PE_{AAS}\right)}$ e $W_{k}^{\left(rake_{AAS}\right)})$ com os delineamentos já apresentados.

Como forma de comparar as propostas de pesos calibrados com os pesos fornecidos em conjunto com a subamostra de Exames Laboratoriais, foram estimados parâmetros populacionais para certas variáveis de interesse, conforme a descrição da Tabela 1. As variáveis com código iniciando com a letra "Z" foram obtidas na fase de coleta de Exames Laboratoriais da PNS 2013. As demais variáveis (código iniciando com a letra "Q" ou "J") foram obtidas na primeira fase de coleta da PNS 2013, e estão disponíveis para os 60.202 participantes.

**Tabela 1:** Características de interesse da subamostra de Exames Laboratoriais da PNS 2013 selecionadas para avaliação das estimativas de calibração.

Código da variável	Descrição da variável	Tipo de parâmetro
Q030	Diabetes (*sim, não, apenas na gravidez*)	Total de prevalência
Q002	Hipertensão (*sim, não, apenas na gravidez*)	Total de prevalência
Q060	Colesterol alto (*sim, não*)	Total de prevalência
Q063	Doença do coração [infarto, angina, insuficiência cardíaca ou outra] (*sim, não*)	Total de prevalência
Q068	Acidente vascular cerebral – AVC ou derrame (*sim, não*)	Total de prevalência
Q120	Câncer (*sim, não*)	Total de prevalência
Q124	Insuficiência renal crônica – IRC (*sim, não*)	Total de prevalência
J058	Dengue (*sim, não*)	Total de prevalência
Z025	Creatinina (mg/dL)	Total e média
Z031	Colesterol total (mg/dL)	Total e média
Z034	Hemoglobina glicosilada – HbA1c (%)	Total e média

Para avaliar as estimativas produzidas com os sistemas de pesos propostos na $W_{k}^{\left(PE_{AAS}\right)}$ e $W_{k}^{\left(rake_{AAS}\right)}$ foram calculadas as 7 medidas apresentadas no Material Suplementar 2. As análises foram realizadas no *software* R^17^ em conjunto com o pacote *survey* ^18^ para incorporar o delineamento de amostragem. O código em R com a especificação do objeto de delineamento (*svydesign*) utilizado nas análises é apresentado no Material Suplementar 3.

## RESULTADOS

### Distribuição dos pesos

As distribuições dos pesos de $W_{k}^{\left(lab\right)}$ e $W_{k}^{\left(PE_{AAS}\right)}$ possuem forma similar, no entanto, os valores que cada sistema de pesos assume é muito distinta, refletindo o que cada um dos sistemas de pesos representa: se, por um lado, $W_{k}^{\left(lab\right)}$ tem como alvo representar a amostra da primeira fase da PNS 2013 (Figura 1A); por outro lado, $W_{k}^{\left(PE_{AAS}\right)}$ tem como objetivo representar a população-alvo da pesquisa, ou seja, adultos brasileiros residentes em domicílios particulares permanentes (Figura 1B). A distribuição de pesos do *raking* apresenta uma forma distinta das demais distribuições de pesos $W_{k}^{\left(lab\right)}$ e $W_{k}^{\left(PE_{AAS}\right)}$ , mas com valores parecidos com os pesos $W_{k}^{\left(PE_{AAS}\right)}$ . Assim, os pesos $W_{k}^{\left(rake_{AAS}\right)}$ também produzem estimativas generalizáveis para a população-alvo da PNS 2013 (Figura 1C).

**Figura 1:** Distribuição dos pesos de (A) pós-estratificação disponibilizados junto aos dados da subamostra de exames laboratoriais da PNS 2013; e de (B) pós-estratificação e (C) *raking* construídos a partir da projeção populacional – subamostra de Exames Laboratoriais, PNS 2013.

### Representatividade dos pós-estratos

Os pesos $W_{k}^{\left(lab\right)}$ apresentam estimativas das proporções das variáveis auxiliares próximas da projeção populacional (Figura S1, Material Suplementar). Em geral, as estimativas com base em $W_{k}^{\left(lab\right)}$ apresentam diferença (erro absoluto) de, no máximo, 0,15%, com exceção das categorias raça/cor parda (0,22%), raça/cor outra (0,42%) e região Nordeste (-0,26%). Já as estimativas obtidas pelos pesos $W_{k}^{\left(PE_{AAS}\right)}$ apresentaram erro absoluto, em geral, também não maior que 0,15%, com exceção das categorias raça/cor branca (0,21%), parda (-0,20%) e outra (0,43%). Com os pesos calibrados pelo método do raking, $W_{k}^{(rake_{AAS})}$ , as estimativas das proporções das variáveis auxiliares coincidem com as projeções populacionais. Resultados semelhantes foram obtidos ao se comparar as estimativas dos totais populacionais das variáveis auxiliares (Tabela S1, Material Suplementar 4). Consideramos calcular o erro relativo (ER) de $W_{k}^{\left(lab\right)}$ , no entanto, é possível notar que as estimativas populacionais oriundas desses pesos são muito diferentes das projeções populacionais, o que resulta em um ER extremamente alto — por isto optamos por não apresentar esses resultados.

### Medidas de avaliação da calibração

As 7 medidas de avaliação dos métodos de calibração são apresentadas na Tabela 2. A média do ER absoluto de calibração foi *M*_1_ = 2,16 para o método de pós-estratificação e *M*_1_ = 0 para o *raking*. Esses resultados já eram esperados, tendo em vista que os totais estimados para as variáveis auxiliares foram, em geral, inferiores aos totais populacionais. A média dos coeficientes de variação dos totais das variáveis auxiliares *M*_2_ = 0% para ambos os métodos indica que estimativas dos totais das variáveis auxiliares produzidos pelos dois métodos são não tendenciosas.

**Tabela 2:** Medidas de desempenho dos métodos de calibração por pós-estratificação e *raking* – subamostra de Exames Laboratoriais, PNS 2013.

Medida de avaliação	$W_{k}^{\left(PE_{AAS}\right)}$	$W_{k}^{\left(rake_{AAS}\right)}$
M1: média do erro relativo absoluto de calibração	2,16	0,00
M2: média dos coeficientes de variação (CV) dos totais das variáveis auxiliares	0,00%	0,00%
M3: proporção dos pesos extremos em relação a 1/3	19,02%	18,81%
M4: proporção dos pesos extremos em relação a 3	4,39%	3,24%
M5: coeficiente de variação dos pesos *g*	91,37%	83,94%
M6: distância *X* ^2^ entre *w* e *d*	13.440,07	11.447,09
M7: eficiência média das estimativas de calibração	1,005	1,018

As medidas *M*_3_ e *M*_4_ (proporção de pesos extremos) indicam a presença de pesos *g* extremos tanto para o método de pós-estratificação quanto para o *raking*. Isso era esperado, tendo em vista a distância entre os pesos assumidos como pesos de delineamento *d*_k_ e os pesos construídos $W_{k}^{\left(PE_{AAS}\right)}$ e $W_{k}^{\left(rake_{AAS}\right)}$ (*M*_6_ = 13.440,07 para a pós-estratificação e *M*_6_ = 11.447,09 para o *raking*). O coeficiente de variação dos pesos *g* também indica essa característica da construção dos pesos calibrados e foi alto para os dois métodos (*M*_5_ = 91,37% para a pós-estratificação e *M*_5_ = 83,94% para o *raking*), apresentando uma leve vantagem para o método *raking*.

A eficiência média das estimativas (*M*_7_) pelos métodos alternativos de calibração para o conjunto de variáveis apresentadas na Tabela 1 indicam que há uma pequena vantagem do método de pós-estratificação em comparação ao *raking*.

### Precisão das estimativas dos parâmetros de interesse

A Tabela 3 apresenta as estimativas para os parâmetros relacionados às características de interesse (listados na Tabela 1), juntamente com os coeficientes de variação obtidos a partir dos três sistemas de pesos calibrados. Observa-se que as estimativas pontuais (totais, prevalências e médias) produzidas pelos três métodos, em geral, são muito próximas. A exceção diz respeito à estimativa dos totais populacionais a partir dos pesos pós-estratificação da própria subamostra de Exames Laboratoriais, os pesos $W_{k}^{\left(lab\right)}$ . Como já observado, tais resultados não são generalizáveis para a população-alvo da PNS 2013. Por fim, as estimativas de coeficiente de variação das estimativas apresentam maior precisão dos métodos de calibração alternativos propostos neste trabalho. Este resultado era esperado, uma vez que a estimação do erro-padrão das estimativas leva em consideração os aspectos do delineamento amostral assumido.

**Tabela 3:** Totais e prevalências (%)‡ estimados e coeficiente de variação (CV%) das características de interesse obtidos a partir dos três sistemas de pesos calibrados — subamostra de Exames Laboratoriais, PNS 2013.

Características de interesse	$W_{k}^{\left(lab\right)}$ (N = 8.952)		$W_{k}^{\left(PE_{AAS}\right)}$ (N = 144.908.922)		$W_{k}^{\left(rake_{AAS}\right)}$ (N = 145.572.210)	
	Estimativa (%)	CV%	Estimativa (%)	CV%	Estimativa (%)	CV%
*Diabetes*	573(7,69)	5,04	9.275.930(7,7)	4,76	9.266.527(7,65)	4,85
*Hipertensão*	1.930(25,91)	2,53	31.236.815(25,92)	2,18	31.171.025(25,75)	2,25
*Colesterol alto*	1.160(15,57)	3,42	18.773.491 (15,58)	3,2	18.842.610(15,56)	3,25
*Doença do coração*	370(4,96)	6,15	5.971.338(4,95)	5,83	6.092.130(5,03)	5,98
*AVC*	146(1,96)	11,57	2.367.716(1,96)	10,47	2.266.318(1,87)	10,3
*Câncer*	158(2,11)	9,87	2.540.009(2,11)	9,45	2.549.079(2,11)	9,57
*IRC*	111(1,48)	12,04	1.785.325(1,48)	11,78	1.815.305(1,5)	11,73
*Dengue*	1.406(18,88)	3,06	22.735.194(18,86)	2,9	22.732.548(18,78)	2,91
*Creatinina (Média, mg/dL)*	0,881	0,44	0,881	0,38	0,882	0,44
*Colesterol total (Média, mg/dL)*	185,031	0,31	185,01	0,29	184,815	0,29
*HbA1c (Média, %)*	5,513	0,23	5,513	0,22	5,51	0,23

‡ Para as características Creatinina, Colesterol total e HbA1c foram estimados média e CV.

Com o propósito de avaliar o desempenho dos métodos de calibração na estimativa em subgrupos da população, foram obtidas estimativas de prevalência de diabetes de acordo com algumas características de interesse (Tabela 4). Mais uma vez, percebe-se que as estimativas pontuais são muito semelhantes para os três sistemas de peso, e que o ganho aparente dos métodos alternativos de calibração se dá nas estimativas de precisão. Os intervalos de confiança de 95% para a prevalência de diabetes nos subgrupos da população produzidos pelos métodos de pós-estratificação $\left(W_{k}^{\left(PE_{AAS}\right)}\right)$ e *raking* $\left(W_{k}^{\left(rake_{AAS}\right)}\right)$ são ligeiramente mais estreitos que aqueles produzidos pelo peso $W_{k}^{\left(lab\right)}$ .

**Tabela 4:** Prevalência de diabetes (em %) e intervalo de confiança de 95% (IC95%) de acordo com subgrupos da população obtidos a partir dos três sistemas de pesos calibrados – subamostra de Exames Laboratoriais, PNS 2013.

Características		$W_{k}^{\left(lab\right)}$		$W_{k}^{\left(PE_{AAS}\right)}$		$W_{k}^{\left(rake_{AAS}\right)}$	
		%	IC95%	%	IC95%	%	IC95%
Idade							
	18 a 29 anos	1,4	0,5–2,4	1,4	0,6–2,3	1,3	0,5–2,0
	30 a 44 anos	2,4	1,6–3,3	2,4	1,6–3,2	2,4	1,6–3,1
	45 a 59 anos	10,8	9,1–12,5	10,8	9,2–12,5	11,1	9,4–12,8
	≥60 anos	18,2	15,9–20,5	18,2	16,0–20,5	17,9	15,6–20,2
Raça/cor							
	Branca	7,3	6,2–8,4	7,3	6,3–8,3	7,3	6,3–8,4
	Preta	9,6	6,8–12,5	9,7	7,1–12,2	9,7	6,9–12,4
	Parda	7,2	6,2–8,3	7,2	6,2–8,2	7,1	6,1–8,2
	Outra	6,0	1,1–10,8	45,5	2,0–8,9	4,6	1,1–8,0
Índice de Massa Corporal – IMC (kg/m²)							
	Baixo peso/normal (IMC<25)	4,2	3,4–4,1	4,2	3,4–5,0	4,3	3,4–5,2
	Sobrepeso (25≤IMC<30)	7,5	6,2–8,7	7,5	6,2–8,7	7,2	6,0–8,4
	Obesidade (IMC≥30)	13,5	11,4–15,5	13,5	11,5–15,4	13,5	11,6–15,5
Plano de Saúde							
	Sim	7,1	5,8–8,4	7,1	5,8–8,3	7,0	5,8–8,3
	Não	7,6	6,8–8,5	7,7	6,8–8,5	7,6	6,7–8,4

## DISCUSSÃO

A Pesquisa Nacional de Saúde em 2013 envolveu a coleta de uma subamostra de exames laboratoriais, importante contribuição para estudos da saúde da população brasileira. As técnicas de amostragem sugerem que um delineamento em duas fases poderia ter sido empregado na subamostra da PNS 2013 para a construção de sistemas de pesos básicos e calibrados. No entanto, dificuldades na coleta da amostra da segunda fase resultaram na não divulgação dos pesos de delineamento em conjunto com os microdados da subamostra de Exames Laboratoriais. Na ausência de pesos básicos de amostragem, os gestores dos dados da subamostra forneceram pesos calibrados pelo método de pós-estratificação.

Neste trabalho, propusemos dois métodos de calibração alternativos com base na pós-estratificação e no *raking*. Os sistemas de pesos obtidos a partir destes métodos apresentaram desempenho concorrente ao sistema de pesos disponibilizado junto aos dados dos Exames Laboratoriais. Em particular, destaca-se que as estimativas obtidas a partir dos sistemas de pesos sugeridos são generalizáveis para a população-alvo da PNS 2013: a população brasileira de adultos residentes em domicílios particulares.

Outro aspecto a ser enfatizado é que os dois métodos de calibração propostos apresentaram maior precisão para as estimativas consideradas neste trabalho. Uma possível explicação para esse comportamento das estimativas é o fato de termos utilizado alguns aspectos importantes, embora de forma suposta, do delineamento do levantamento por amostragem, que resultam no cálculo adequado da estimativa da variância das estimativas de parâmetros de interesse.

Ao utilizar medidas de avaliação do desempenho dos métodos de calibração, os dois sistemas de pesos sugeridos apresentaram um bom desempenho, como uma vantagem para os pesos baseados no *raking*. Alguns trabalhos anteriores apontam para as mesmas conclusões^19–22^. No entanto, é preciso salientar que medidas referentes à comparação entre os pesos calibrados e "pré-calibrados" identificam uma distância muito grande entre essas duas ponderações. É provável que esse comportamento seja observado pelo fato de termos assumido um delineamento de amostragem aleatória simples a partir da amostra da primeira fase da PNS 2013 para os dados da subamostra de Exames Laboratoriais, enquanto os pós-estratos foram construídos a partir das projeções da população brasileira.

Como pode ser observado, a subamostra de Exames Laboratoriais da PNS 2013 poderia ser vista como resultado de um delineamento de amostragem em duas fases^16^. No entanto, as probabilidades de seleção (ou, de forma análoga, os pesos básicos de amostragem) não foram disponibilizadas no arquivo de microdados dos Exames Laboratoriais, e assim, as vantagens de um delineamento em duas fases não puderam ser empregadas. A disponibilização dos pesos básicos de delineamento da subamostra de Exames Laboratoriais pode auxiliar estatísticos e epidemiologistas a construir sistemas de pesos calibrados de tal forma a melhorar o desempenho dos estimadores de calibração.

Com respeito à estimativa de totais populacionais (*e.g.*, o total de indivíduos com diabetes), os dois métodos propostos por este artigo fornecem estimativas para a população-alvo da PNS 2013, generalizando os resultados para a população de adultos brasileiros. Já o sistema de pesos fornecidos em conjunto com os dados da subamostra de Exames Laboratoriais resultou em estimativas para a amostra da PNS 2013. Embora seja possível obter estimativas para a população-alvo da PNS de forma indireta, multiplicando-se a proporção amostral pelo tamanho da população, é preferível que os métodos, em conjunto com os *softwares* adequados, forneçam diretamente essas estimativas para evitar erros de interpretação dos resultados.

A abordagem adotada neste artigo foi a da ponderação por meio de métodos de calibração. Algumas limitações do nosso trabalho são inerentes à referida abordagem, por exemplo, a aplicação de pesos para estimativas mais complexas, como coeficientes de regressão; a dificuldade em avaliar os erros-padrões das estimativas ponderadas; e as decisões envolvidas na criação de sistemas de pesos^23^. Ainda assim, recomendamos que estimativas ponderadas sejam utilizadas no lugar de estimativas não ponderadas a partir de dados de inquéritos populacionais de delineamento de amostragem complexo. Uma outra limitação deste trabalho foi a suposição de certos aspectos do delineamento empregado para a coleta dos dados da subamostra de Exames Laboratoriais da PNS 2013. Como já enfatizado na literatura, as análises devem considerar os aspectos relevantes do plano amostral^14^, e a disponibilização de tais aspectos referentes ao plano da subamostra de Exames Laboratoriais da PNS proporcionaria aos pesquisadores maior precisão às estimativas das quantidades populacionais de interesse.

Finalizamos com duas sugestões para os amostristas e gestores das Pesquisas Nacionais de Saúde que serão utilizadas por outros pesquisadores. Primeiro, que todos os pesos referentes ao delineamento da PNS sejam documentados e incluídos nos arquivos de dados de uso público. Isso possibilitaria que os usuários construíssem estimativas a partir dos pesos básicos ou calibrados, utilizando as suas próprias variáveis auxiliares. A correta precisão das estimativas poderia ser obtida com a utilização adequada de *softwares* de análise de dados oriundos de planos amostrais complexos.

A nossa segunda recomendação diz respeito ao chaveamento das informações obtidas na amostra e subamostra (de Exames Laboratoriais) da PNS 2013. Tal informação permitiria a exploração de métodos como a imputação de dados para obter estimativas mais precisas. Dada a grande relevância destes dados para a pesquisa em epidemiologia e saúde coletiva, os métodos mais adequados devem ser empregados na análise dos mesmos.
